# Evaluation of the Intestinal Anti-Inflammatory Property of *Spondias mombin* L. in an Experimental Animal Model of Colitis

**DOI:** 10.3390/pharmaceutics18060723

**Published:** 2026-06-11

**Authors:** Yasmim Vilarim Barbosa, Maria Elaine Cristina Araruna, Maria Lorenna Pessoa Fonsêca, Francisco José Batista de Lima Júnior, Cassiano Francisco Weege Nonaka, Paulo César Dantas da Silva, José Elizandro Batista de Oliveira, Bruna Larissa Barbosa de Lira, Thássia Borges Costa, Vanda Lucia dos Santos

**Affiliations:** 1 Programa de Pós-Graduação em Ciências Farmacêuticas, Universidade Estadual da Paraíba, Campina Grande 58429-500, PB, Brazil; jose.elizandro@aluno.uepb.edu.br (J.E.B.d.O.); vandalsantos@servidor.uepb.edu.br (V.L.d.S.); 2 Laboratório de Ensaios Farmacológicos, Universidade Estadual da Paraíba, Campina Grande 58429-500, PB, Brazil; elaine.araruna@gmail.com (M.E.C.A.); marialorennaaa@gmail.com (M.L.P.F.); franzebatista94@gmail.com (F.J.B.d.L.J.); brunalarissa31@gmail.com (B.L.B.d.L.); thassiacosta5@gmail.com (T.B.C.); 3 Departamento de Farmácia, Universidade Estadual da Paraíba, Campina Grande 58429-500, PB, Brazil; 4 Centro Universitário Unifacisa, Campina Grande 58408-326, PB, Brazil; 5 Departamento de Odontologia, Universidade Estadual da Paraíba, Campina Grande 58429-500, PB, Brazil; cassiano.nonaka@servidor.uepb.edu.br; 6 Laboratório de Avaliação e Desenvolvimento de Biomateriais do Nordeste (CertBio), Universidade Estadual da Paraíba, Campina Grande 58429-500, PB, Brazil; paulodantas@servidor.uepb.edu.br

**Keywords:** inflammatory bowel disease, *Spondias mombin* L., ulcerative colitis

## Abstract

**Background/Objectives**: Inflammatory bowel diseases (IBD) are conditions of the gastrointestinal tract with treatments linked to side effects and relapses. *Spondias mombin* L. is a species with anti-inflammatory action, but there is little information in the literature about its application in the treatment of IBD. The aim of this study was to evaluate the intestinal anti-inflammatory activity of hydroalcoholic extract of *Spondias mombin* L. (HE*Sm*) in a rat model of ulcerative colitis (UC). **Methods**: Hydroalcoholic extract was obtained using the turboextraction technique, followed by identification of its major components using ultra-high performance liquid chromatography (UFLC). Intestinal anti-inflammatory activity was evaluated in an acute model of UC induced by 2,4,6-trinitrobenzenesulfonic acid (TNBS). Groups received a vehicle, prednisolone (2 mg/kg), or HE*Sm* (125, 250 or 500 mg/kg) before and after UC induction. Ulcerated area, score, and intestinal weight/length ratio were analyzed. Histopathological analysis and the extract’s effect on the contractility of the intestinal segment were carried out. **Results**: UFLC identified the presence of quercetin, a flavonoid widely cited for the species. At doses of 125, 250, and 500 mg/kg, the extract reduced areas of injury by 86.82, 92.67, and 85.06%, respectively, compared to the control, in addition to reducing scores and weight/length ratio of the colons. Histopathological analysis confirmed the results. In contractility, the extract at the highest concentration tested reduced the response of the muscarinic agonist carbamylcholine to 53.7 ± 4.2% of control contraction. **Conclusions**: Results demonstrate the species’ ability to reduce injuries caused by colitis, suggesting its potential to contribute to the clinical management of IBD in the future.

## 1. Introduction

Inflammatory bowel diseases (IBD) are conditions of the gastrointestinal tract associated with genetic, immunological, and environmental factors, with Crohn’s disease and UC being the most typical conditions in this spectrum [1,2]. Inflammatory bowel diseases have a high prevalence, reaching a total of 3.8 million cases worldwide in 2021, which highlights their prevalence in the population [3].

Current treatment includes the use of anti-inflammatory drugs and immunosuppressants to relieve symptoms and minimize disease progression and complications. However, the long course of the disease, the costs, and the adverse reactions associated with the medications ultimately lead to low disease remission rates [4,5]. Although they alleviate symptoms during active disease, these conventional treatments are not sufficiently effective in promoting remission and preventing relapses [6].

Over the past 50 years, advances have been made in the pharmaceutical industry due to the discovery of several biologically relevant drugs produced using plant-based raw materials. This has prompted the population to increasingly seek plant-based drugs to treat and prevent various diseases [7,8,9].

Among plant species, *Spondias mombin* L. (Anacardiaceae) stands out, a species popularly known as cajazeira, rich in phenolic compounds [10,11] that promote scientifically proven pharmacological activities for the species, including antioxidant [12], anti-inflammatory [13], and antiulcerogenic [14] activities.

However, studies demonstrating the anti-inflammatory action of *Spondias mombin* L. in the intestine are scarce. Therefore, this study aims to evaluate the intestinal anti-inflammatory potential of the hydroalcoholic extract of *Spondias mombin* L. (HE*Sm*), contributing to scientific knowledge about the species and consequently enabling the development of new pharmacological options that are safer and more effective than the drugs currently available for the treatment of IBD.

## 2. Materials and Methods

### 2.1. Plant Material and Extract Preparation

The leaves of *Spondias mombin* L. were collected during February and March 2023 at a private site in the city of Matinhas, in the semi-arid region of Paraíba (7°07′20.52″ S 35°46′16.99″ W). The species voucher (number 003238/2021) was deposited at the Manuel de Arruda Câmara Herbarium (ACAM) at the State University of Paraíba. The plant material was dehydrated and subjected to turbo extraction, alternating between four 30 s cycles of agitation and four minutes of rest, with a ratio of 100 g of plant material to 1000 mL of 70% (*v*/*v*) ethyl alcohol. The material was then filtered, transferred to a rotary evaporator (45 °C) to remove the ethanol, and then dried in an oven at 40 °C for 72 h until completely dry, obtaining at the end of the process the hydroalcoholic extract of *Spondias mombin* L. (HE*Sm*).

### 2.2. Ultra Fast Liquid Chromatography (UFLC) Analysis

The method was developed to obtain the chromatographic profile of the extract. A Shimadzu^®^ ultra-high performance liquid chromatograph (UFLC) equipped with two LC-20AD pumps, SIL-20A-HT automatic injector, CTO-20A column oven, SPD-20A UV/Vis detector, CBM-20A controller (Shimadzu, Tokyo, Japan), and computerized automatic integrator with LC Solution^®^ 1.25 software was used. The stationary phase consisted of an ACE 5 C18 analytical column (i.d. 4.6 mm × 250 mm, 5.0 µm; Avantor ACE Generix, Kent, UK). The mobile phase consisted of water (solvent A) and acetonitrile (solvent B) under linear gradient elution conditions ranging from 5% to 100% solvent B over 60 min. The mobile phase consisted of a gradient elution of acetonitrile:water (10:90; *v*/*v*). The analyses were performed at a controlled temperature (40 °C), with a flow rate of 1.0 mL.min^−1^ and an injection volume of 10 µL, with a wavelength of 256 nm. The peaks of interest were identified by co-injection with the reference substance (hydrated quercetin, batch: 8TBC5253V, 95%, Sigma Aldrich^®^, St. Louis, MO, USA) through fortification of the extract with solutions of known concentration of the standard: 10 ug/mL, 15 µg/mL, and 30 µg/mL [15].

### 2.3. Biological Activity

#### 2.3.1. Animals

Male Wistar rats (Rattus norvegicus), *n* = 70 (180–250 g; 7–8 weeks old), supplied by the bioterism center of the State University of Paraíba, were used. The animals were kept in plastic cages, with a 12 h light–dark cycle, controlled temperature, and ad libitum water and feed. The experiments followed the guidelines of the National Council for the Control of Animal Experimentation (CONCEA) and the Ethics Committee on the Use of Animals (CEUA), with approval protocol N° 041/2023. Every effort was made to reduce the number of animals used, and procedures from the beginning of the study until the moment of euthanasia were performed to avoid suffering and reduce discomfort and pain in the animals.

#### 2.3.2. Evaluation of the Effects of HES*m* on Colitis Caused by 2,4,6-Trinitrobenzenesulfonic Acid (TNBS)

The experimental protocol described by Morris et al. [16] was conducted with modifications. After fasting for 24 h, the rats were anesthetized with 2% xylazine hydrochloride and 10% ketamine hydrochloride for rectal administration of TNBS (10 mg per animal dissolved in 0.25 mL of 50% ethanol) with the aid of a 2 mm diameter probe inserted approximately 8 cm into the rectum. The animals were kept upside down for 10 min. Each group of animals was pretreated orally with vehicle (saline solution 10 mg/kg—colitic group), 2 mg/kg prednisolone (standard control group), HE*Sm* in the doses of 125, 250, or 500 mg/kg (according on the in vivo study by Araruna et al. [14], with adaptations) 48, 24, and 1 h before TNBS administration and 24 h after inflammation induction. Forty-eight hours after TNBS administration, the rats were euthanized intraperitoneally with a combination of 2% xylazine hydrochloride and 10% ketamine hydrochloride anesthetics.

Colonic segments were collected and photographed to quantify the ulcerative lesion area (ULA) using ImageJ 1.54g software and determine the macroscopic score. The weight-to-length ratio of the entire colon was also evaluated. An untreated, non-colitic group was added to the experiment, whose animals were not subjected to inflammation induction. The intestinal lesion score was evaluated according to the scale described by Bell et al. [17]: (1) focal hyperemia, no ulcer; (2) ulceration, no hyperemia/thickening of the intestinal wall; (3) ulceration, inflammation in one location; (4) ulceration, inflammation in two or more locations; (5) severe lesion > 1 cm; (6–10) severe lesions > 2 cm.

The percentage of injury inhibition (%) was calculated as described below (1):

(1) $$\mathrm{Injury}\;\mathrm{inhibition}\;\left(\%\right)\;=\;\frac{\mathrm{Negative}\;\mathrm{control}\;\mathrm{ALU}\;-\;\mathrm{Treated}\;\mathrm{group}\;\mathrm{ALU}}{\mathrm{Negative}\;\mathrm{control}\;\mathrm{ALU}}\;\times \;100$$

To calculate the weight/length ratio, the following formula was used (2):

(2) $$\frac{\mathrm{Weight}\;\mathrm{of}\;\mathrm{the}\;\mathrm{colon}\;(g)}{\mathrm{Length}\;\mathrm{of}\;\mathrm{the}\;\mathrm{colon}\;(\mathrm{cm})}$$

After determining ALU, tissue samples from colon lesions were collected and stored in 10% buffered formaldehyde solution for histopathological analysis.

#### 2.3.3. Histopathological Analysis

Fragments of colonic tissue from all groups were fixed in 10% buffered formaldehyde for 24 h and embedded in histological paraffin. Tissue sections (5 µm thick) were obtained and stained with hematoxylin and eosin staining. Next, the slides were scanned to create high-resolution digital images (MoticEasyScan Pro 6, Motic Inc., Richmond, BC, Canada) and viewed in the DSAssistant program (Motic Inc., Richmond, BC, Canada).

In the histopathological analysis, two parameters were analyzed: lesion length and lesion score.

The lesion length was determined in micrometers (µm) by averaging all lesions found in each colon (*n* = 3) per treated group.

The methodology described by Lee et al. [18] was used to determine the lesion score. The assessment included reports of leukocyte infiltration, lesion extent, and crypt loss. The severity of inflammation was scored using a scale from 0 to 3 (0, no inflammation; 1, mild inflammation; 2, moderate inflammation; and 3, severe inflammation), as well as the extent of the lesion (0, no lesion; 1, mucosal lesion; 2, mucosal and submucosal lesion; and 3, transmural lesion). Crypt damage was scored using a scale from 0 to 4 (0, no damage; 1, basal third damaged; 2, two basal thirds damaged; 3, only the superficial epithelium was intact; and 4, loss of the entire crypt and epithelium). Each value was multiplied by an extent index ranging from 1 to 4, which reflected the amount of involvement of each section (1, 0 to 25%; 2, 26 to 50%; 3, 51 to 75%; and 4, 76 to 100%). At least three sections from each colon (*n* = 3) were analyzed.

### 2.4. Evaluation of HESm on the Contractility of Isolated Colon Segments

The experimental protocol described by Lima et al. [19] was used in Wistar rats (*n* = 3), which were euthanized by lethal dose with anesthetics (2% Xylazine and 10% Ketamine). Subsequently, approximately 5 cm segments of colon were removed and mounted longitudinally in an isolated organ bath (Insight Ltd., Ribeirão Preto, São Paulo, Brazil) immersed in Tyrode’s physiological solution at 37 °C, under constant aeration with ambient air and basal tension of 1 g. The experimental protocol consisted of adding a contractile stimulus with carbamylcholine in the presence of increasing concentrations of HE*Sm* to identify the influence of the extract on the contraction pattern of this muscarinic agonist.

First, stimulation was performed by raising the potassium (K^+^) concentration to 60 mM in order to verify tissue viability. Next, with the renewed physiological solution, the tissue was exposed to stimulation with carbamylcholine (1 µM), which served as a reference contraction. Subsequently, the same carbamylcholine stimulation was repeated with prior addition (5 min) of the extract at concentrations of 10, 100, and 1000 μg/mL, with renewal of the physiological solution at each concentration tested. The contraction amplitudes were expressed in relation to the percentage of carbamylcholine.

### 2.5. Statistical Analysis

Results were expressed as mean ± standard deviation (SD) or mean ± standard error of the mean (SEM) for parametric values, or as median (minimum value − maximum value) for nonparametric data. One-way ANOVA (parametric data) or Kruskal–Wallis test (nonparametric data) was performed, followed by Dunnet and Tukey or Dunn post-tests, respectively. Results were significant when *p* < 0.05. Data were analyzed using GraphPad^®^ 5.0 software.

## 3. Results

### 3.1. Ultra Fast Liquid Chromatography (UFLC)

**Figure 1:**
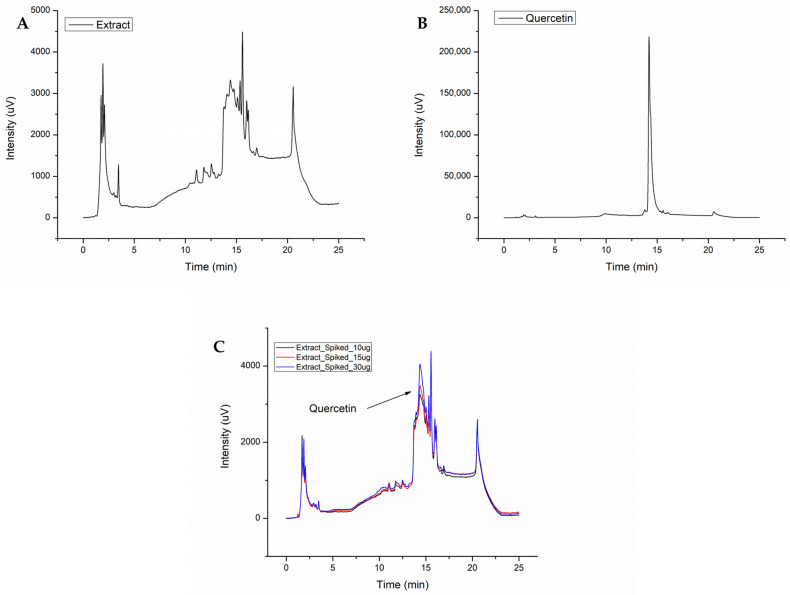
Chromatographic analysis of *S. mombin* L. extract. Chromatogram of the HE*Sm* detected at 256 nm (**A**), peak and retention time of the quercetin standard (Tr = 14.3) (**B**), and superimposition of the curves of the HE*Sm* fortified with the quercetin standard at different concentrations (**C**).

When analyzing the chromatographic profile of the extract, a peak indicative of the flavonoid quercetin was suggested (Figure 1A), a metabolite widely mentioned in the species *S. mombin* L., since the retention time of 14.3 min recorded for the extract was the same as that recorded for the quercetin standard (Figure 1B).

Quercetin was tentatively identified in the extract based on retention time similarity and proportional peak enhancement after co-injection with quercetin standard solutions at concentrations of 10, 15, and 30 μg/mL (Figure 1C). The progressive increase in the intensity of the corresponding chromatographic peak after spiking supported the preliminary identification of the compound.

### 3.2. Effect of S. mombin L. on Acute Intestinal Inflammation in a TNBS-Induced Colitis Model

The area of ulcerative lesion, the percentage of injury inhibition, the lesion score, and the weight/length ratio of the colons were evaluated 48 h after TNBS administration, as shown in Table 1.

**Figure 2:**
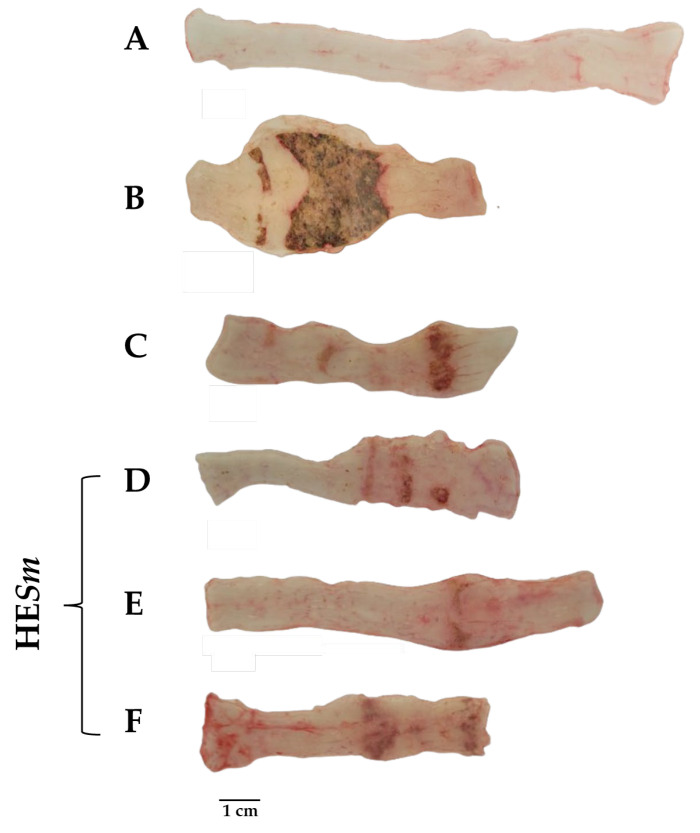
Representative image of the colons of rats from the non-colitic group (**A**); colitic group (**B**); positive control (prednisolone) (**C**) and HE*Sm* at doses of 125, 250 and 500 mg/kg (**D**–**F**) submitted to the TNBS-induced acute ulcerative colitis model.

TNBS administration in the colitic group developed extensive lesions and intense signs of inflammation with macroscopic ulcerative areas, accompanied by an increase in the weight/length ratio of the colonic segment, compared to the non-colitic group (*p* < 0.001) as shown in Table 1. Representative images of the colons are shown in Figure 2.

HES*m* at doses of 125, 250, and 500 mg/kg significantly reduced (*p* < 0.001) the area of ulcerative lesions by 86.82, 92.67, and 85.06% compared to the control, respectively. Prednisolone, used as a positive control, also significantly reduced the lesion area by 88.33% compared to the control (*p* < 0.001). Treatment with HES*m* did not show a dose-dependent effect, since there was no statistically difference between the tested extract doses, indicating that the lowest dose is sufficient to promote the anti-inflammatory effect in the intestine.

The results showed that all doses of the extract, 125, 250, and 500 mg/kg, significantly reduced (*p* < 0.001) the lesion score by 2.2 ± 0.4, 0.7 ± 0.5, and 2.0 ± 0.7, respectively, when compared to the control, indicating a regression of the intestinal inflammatory process.

For the colons analyzed, a shortening in length was identified for the colitic group, as shown previously in Figure 2, while all doses of HE*Sm* reduced the ratio when compared to the control, with doses of 250 and 500 mg/kg presenting the lowest ratios (113.8 and 131. 9 mg/cm, respectively), when compared to the group treated with prednisolone (138.8 mg/cm), demonstrating the potential of the extract in comparison to the standard drug.

### 3.3. Histopathological Analysis

**Figure 3:**
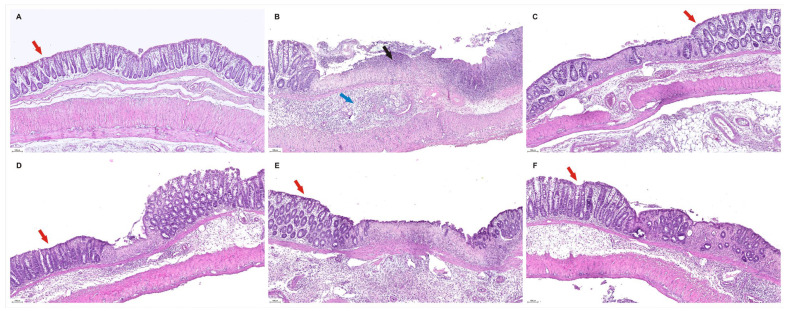
Microscopic effects of the colon of rats subjected to the TNBS-induced acute ulcerative colitis model and treated or not with *S. mombin* L. Representative photomicrographs of the colon of animals in the experimental groups: HE staining—Non-colitic (**A**), Colitic (Saline solution) (**B**), prednisolone 2 mg/kg (**C**), and HE*Sm* 125, 250 and 500 mg/kg (**D**–**F**). Red arrows (intact epithelium), black arrow (destroyed crypts), blue arrow (inflammatory cells).

Histological sections of colons belonging to the non-colitic, prednisolone, and HE*Sm* groups at doses of 125, 250, and 500 mg/kg (Figure 3A, 3C, 3D, 3E, and 3F, respectively) demonstrate the integrity of the epithelium (red arrows) and goblet cells. In the colitic group (Figure 3B), intense destruction of the crypts (black arrow) was identified, with exposure of the connective tissue and high migration of inflammatory cells (blue arrow), such as neutrophils, lymphocytes, plasma cells, and macrophages, crossing the innermost layer of the GI tract wall, the muscular layer.

**Figure 4:**
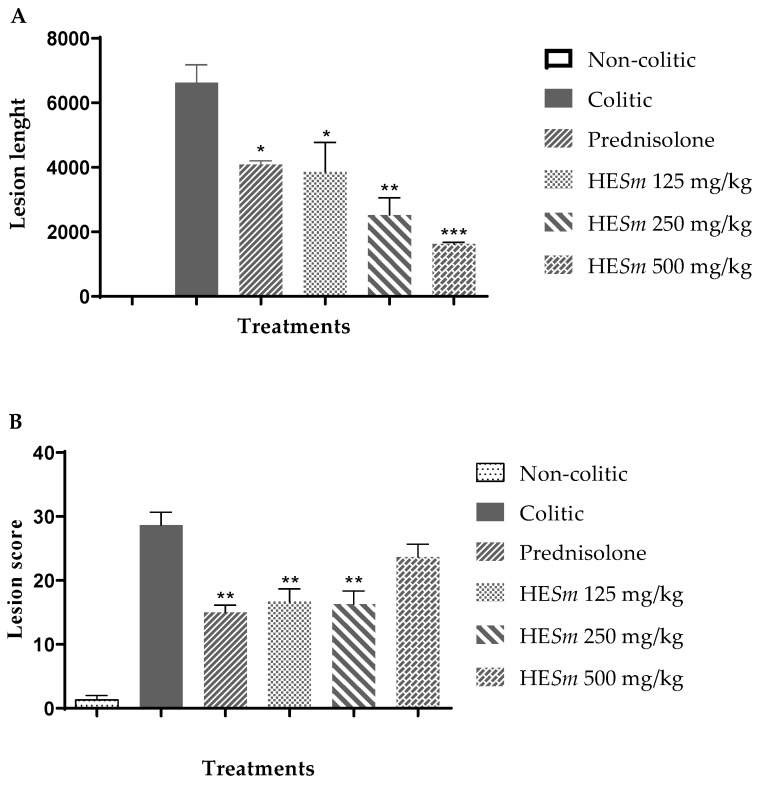
Lesion length (**A**) and Lesion score (**B**) of *S. mombin* L. extract. Data expressed as mean ± SD. For parametric data, we used One Way ANOVA with Dunnett’s or Tukey’s post-tests. * *p* < 0.05, ** *p* < 0.01, *** *p* < 0.001 compared to colitic. HE*Sm* = Hydroalcoholic extract of *Spondias mombin* L.

All tested doses of the extract significantly reduced the lesion length when compared to the colitic group (Figure 4A). At doses of 125 and 250 mg/kg, HES*m*, like prednisolone, significantly reduced the lesion score when compared to the colitic group, as shown in Figure 4B.

The 500 mg/kg dose did not significantly reduce the lesion score, contrasting with the ability of this dose to reduce TNBS-induced lesions macroscopically and microscopically. As identified in the histopathological images (Figure 3F), the 500 mg/kg dose reduces the lesion length; however, the lesion depth is greater, with TNBS generating an intense level of aggression with transmural cellular infiltrate, which justifies the results obtained.

### 3.4. In Vitro Evaluation of Intestinal Segment Contractility

**Figure 5:**
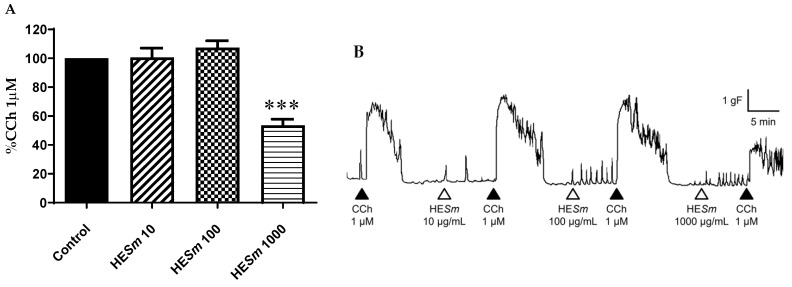
In vitro evaluation of the hydroalcoholic extract of *S. mombin* L. on the contractility of colitic segments (**A**) and its respective experimental tracing (**B**). Data are expressed as mean ± SEM. One-way ANOVA with Dunnett’s post-tests was used. *** *p* < 0.001 compared to the control. HE*Sm* = Hydroalcoholic extract of *Spondias mombin* L.

Considering the more irregular pattern of spontaneous colon activity, a protocol was chosen involving the evaluation of the extract’s ability to interfere with the contraction pattern of the muscarinic agonist carbamylcholine. The results obtained for analysis are shown in the graph in Figure 5A and in the respective experimental plot in Figure 5B.

It was possible to identify that at the highest concentration tested, 1000 μg/mL, the extract was able to reduce the carbamylcholine response to 53.7 ± 4.2% of the control contraction (*n* = 6, *p* ˂ 0.05), clearly reducing the amplitude of the contraction, as identified in the experimental trace above, indicating that the extract has the potential to alter the pattern of intestinal motility.

## 4. Discussion

*Spondias mombin* L. is a plant species belonging to the Anacardiaceae family, widely found in Brazil, mainly in the North, Northeast, and Central-West regions. It is composed of secondary metabolites such as flavonoids, saponins and tannins, which are responsible for the different pharmacological activities attributed to the species [20,21].

Currently, several techniques have been used in an attempt to evaluate plant-based materials, such as high-performance liquid chromatography, which allows the identification of chemical markers and the comparison of the chemical profiles of herbal medicines [22].

In our study, through chromatographic analysis, the flavonoid quercetin was identified in HE*Sm*. This metabolite belongs to the flavonol class, a subclass of flavonoids, and is attributed with various pharmacological activities, including antioxidant and immunomodulatory properties [23].

Quercetin has already been widely reported in the literature for *S. mombin*, as in studies by Da Silva et al. [24], who identified quercetin in the hydro-methanolic extract of the leaves, and in the study by Rey-Blanes et al. [25], which revealed the presence of quercetin dipentoxide in the hydroalcoholic extract of the leaves. These data reinforce the presence of this metabolite in our study.

Different experimental methodologies are used to evaluate IBD, including models of colitis induced by chemical agents such as TNBS. Ulcerative colitis is a gastrointestinal tract disorder that presents a pattern of diffuse and continuous lesions, which can extend from the rectum to the colon, affecting the mucosal and submucosal layers of the large intestine [26,27]. The main symptoms related to UC include diarrhea, usually accompanied by blood, abdominal pain, and weight loss [28].

The pathophysiology of UC, together with that of Crohn’s disease, is complex and not fully understood, but it is correlated with different factors, including poor lifestyle, environmental factors, dysfunctional intestinal microbiota, genetic factors, and an inadequate immune response [29,30].

Based on this, the methodology selected to evaluate intestinal anti-inflammatory activity in this study was colitis induced by the chemical agent TNBS. This model induces transmural colitis in animals and is an extremely widely used method because it mimics the acute and chronic stages of IBD [31].

TNBS stimulates an immune response mediated by type 1 helper T cells (Th1), characterized by infiltration of CD4 cells, neutrophils, and macrophages in the lamina propria and promotes cytokine secretion, mainly tumor necrosis factor and interleukin 12 [32].

Macroscopically, a significant reduction in the lesion area was observed in the groups treated with HE*Sm* at all doses tested (Figure 2). This reduction was also observed in the positive control group (prednisolone) when compared to the negative control, which presented an extensive lesion area, increased edema and organ weight, and shortening of the colitic segment.

Statistical analysis confirmed that all tested doses of HES*m*, 125, 250, and 500 mg/kg, significantly reduced the areas of injury caused by TNBS, with no statistically significant difference between doses, indicating the species’ potential to reduce the intestinal inflammatory process characteristic of UC.

These data corroborate the only study in the literature using *S. mombin* L. in an experimental colitis model, developed by Agbaje, Sabo, and Ujomu [33], using aqueous extract from the leaves of the species in a subacute model of acetic acid-induced UC at doses of 50, 100, and 200 mg/kg, in which they observed a significant reduction (*p* < 0.05) in the ulcerative lesion index, of 63.5, 38.4, and 28.8%, respectively.

The weight/length ratio of the colons is an important parameter to analyze, since it can be elevated during the course of inflammation due to the formation of edema and contractions of the muscle layer, which cause changes in the weight and length of these organs and, consequently, in inflamed colons, lead to an increase in this ratio [34,35].

All doses of HE*Sm* reduced the weight/length ratio when compared to the control, as well as the group treated with prednisolone, demonstrating the anti-inflammatory effect of the extract in contributing to the reduction in parameters highly involved in intestinal injuries.

Histopathological analysis can provide valuable insights into the possible therapeutic action mechanisms of plant extracts. The cellular infiltration of neutrophils, macrophages, lymphocytes, and plasma cells identified in histopathology images, combined with the overproduction of pro-inflammatory cytokines, are factors that contribute to mucosal rupture and the consequent formation of lesions characteristic of colitis [36].

At doses of 125 and 250 mg/kg, the groups treated with HE*Sm* exhibited lower leukocyte infiltration, lesion extent, and crypt damage when compared to the colitic group. Moreover, all doses of HE*Sm* significantly reduced the lesion length compared to the colitic group. These findings highlight the anti-inflammatory effects of HE*Sm* and also contributes in understanding the extract’s mechanisms of action.

In the gastrointestinal tract, the outer muscle layer consists of two layers of smooth muscle: the outer longitudinal layer, which shortens the tube when contracted, and the inner circular layer, which decreases the diameter of the lumen when contracted [37]. The contraction patterns represented by these layers are important in controlling gastrointestinal motility and are altered in patients with IBD, since patients affected by these diseases have relatively frequent motility disorders.

The role of calcium (Ca^2+^) is also very important in ensuring intestinal motility. Smooth muscle contraction is regulated by the amount of Ca^2+^ that enters the smooth muscle fiber. This occurs through the role of interstitial Cajal cells (ICC), which generate slow wave potentials that, upon reaching the threshold, promote the opening of voltage-dependent Ca^2+^ channels in the muscle fiber, allowing Ca^2+^ to enter and muscle contraction to occur [38].

At the highest concentration tested, HE*Sm* reduced the contraction pattern of the muscarinic agonist carbamylcholine in longitudinally mounted colonic tissue. Carbamylcholine is a muscarinic agonist responsible for mobilizing calcium and promoting muscle contraction. This is possible because it promotes extracellular calcium recruitment and also releases intracellular calcium from the sarcoplasmic reticulum through signaling with the second messenger, inositol 1,4,5-triphosphate (IP3), and consequently promotes muscle contraction [39,40].

Patients with moderate colitis usually have increased low-amplitude peristaltic contractions, which can contribute to the onset of diarrhea due to accelerated colonic transit [41].

Based on this, our findings indicate that when exposed to high concentrations of the extract, colonic tissue may show a reduced contractile response to cholinergic stimulation, which physiologically results from parasympathetic action. This decrease may be useful in patients with IBD, as it may have a constipating effect and reduce the diarrhea characteristic of these disorders.

## 5. Conclusions

The results demonstrated the in vivo anti-inflammatory effect of the hydroalcoholic extract of *Spondias mombin* L. leaves on the intestine, based on its ability to reduce the damage caused by UC. This study presents, for the first time, histopathological data on the anti-inflammatory activity of this species on the intestine, confirming the macroscopic results obtained. In vitro, the extract’s potential to modulate the amplitude of colon contractions at high concentrations was observed, which may favor a useful constipating effect in patients with IBD. These data demonstrate the potential of the species to promote efficacy in the management of inflammatory bowel diseases and to contribute to the pharmacotherapy of these disorders in the future, reducing the damage caused to patients by conventional treatments.

## Figures and Tables

**Table 1:** Anti-inflammatory effect of *S. mombin* L. in a TNBS-induced colitis model.

Treatments	Dose (mg/kg)	Ulcerative Area (mm^2^)	Injury Inhibition (%)	Injury Score	Weight/Length (mg/cm)
Non-colitic	-	ND	ND	ND	93.3 ± 13.5
Colitic	10	38.48 ± 10.63 ^###^	-	7.4 ± 1.1 ^###^	251.4 ± 21.4 ^###^
Prednisolone	2	4.49 ± 1.96 ***	88.33% ***	1.2 ± 0.7 ***	138.8 ± 15.5 ***
HE*Sm*	125	5.07 ± 1.12 ***	86.82% ***	2.2 ± 0.4 ***	150.4 ± 9.7 ***
HE*Sm*	250	2.82 ± 1.17 ***	92.67% ***	0.7 ± 0.5 ***	113.8 ± 14.0 ***
HE*Sm*	500	5.75 ± 3.13 ***	85.06% ***	2.0 ± 0.7 ***	131.9 ± 16.0 ***

Data are expressed as mean ± SD. For parametric data, we used One-Way ANOVA with Dunnett or Tukey post-tests. *** *p* < 0.001 compared with colitic group; ^###^ *p* < 0.001 compared with non-colitic group. (*n* = 6). ND = not detectable; HE*Sm* = Hydroalcoholic extract of *Spondias mombin* L.

## Data Availability

The original contributions presented in this study are included in the article. Further inquiries can be directed to the corresponding author.
